# Generalizable Beat-by-Beat Arrhythmia Detection by Using Weakly Supervised Deep Learning

**DOI:** 10.3389/fphys.2022.850951

**Published:** 2022-03-22

**Authors:** Yang Liu, Qince Li, Runnan He, Kuanquan Wang, Jun Liu, Yongfeng Yuan, Yong Xia, Henggui Zhang

**Affiliations:** ^1^ School of Computer Science and Technology, Harbin Institute of Technology (HIT), Harbin, China; ^2^ Peng Cheng Laboratory, Shenzhen, China; ^3^ School of Physics and Astronomy, The University of Manchester, Manchester, United Kingdom; ^4^ Key Laboratory of Medical Electrophysiology of Ministry of Education and Medical Electrophysiological Key Laboratory of Sichuan Province, Institute of Cardiovascular Research, Southwest Medical University, Luzhou, China

**Keywords:** cardiac arrhythmia, electrocardiogram, heartbeat classification, weakly supervised learning, generalization ability

## Abstract

Beat-by-beat arrhythmia detection in ambulatory electrocardiogram (ECG) monitoring is critical for the evaluation and prognosis of cardiac arrhythmias, however, it is a highly professional demanding and time-consuming task. Current methods for automatic beat-by-beat arrhythmia detection suffer from poor generalization ability due to the lack of large-sample and finely-annotated (labels are given to each beat) ECG data for model training. In this work, we propose a weakly supervised deep learning framework for arrhythmia detection (WSDL-AD), which permits training a fine-grained (beat-by-beat) arrhythmia detector with the use of large amounts of coarsely annotated ECG data (labels are given to each recording) to improve the generalization ability. In this framework, heartbeat classification and recording classification are integrated into a deep neural network for end-to-end training with only recording labels. Several techniques, including knowledge-based features, masked aggregation, and supervised pre-training, are proposed to improve the accuracy and stability of the heartbeat classification under weak supervision. The developed WSDL-AD model is trained for the detection of ventricular ectopic beats (VEB) and supraventricular ectopic beats (SVEB) on five large-sample and coarsely-annotated datasets and the model performance is evaluated on three independent benchmarks according to the recommendations from the Association for the Advancement of Medical Instrumentation (AAMI). The experimental results show that our method improves the *F*_1_ score of supraventricular ectopic beats detection by 8%–290% and the F1 of ventricular ectopic beats detection by 4%–11% on the benchmarks compared with the state-of-the-art methods of supervised learning. It demonstrates that the WSDL-AD framework can leverage the abundant coarsely-labeled data to achieve a better generalization ability than previous methods while retaining fine detection granularity. Therefore, this framework has a great potential to be used in clinical and telehealth applications. The source code is available at https://github.com/sdnjly/WSDL-AD.

## 1 Introduction

Cardiac arrhythmia has become one of the leading causes of morbidity and mortality worldwide (Roger et al., 2012). Ambulatory electrocardiogram (ECG) monitoring with prolonged duration (several days or weeks) provides critical information for early detection and treatment of arrhythmias, especially for transient and asymptomatic arrhythmias (Sana et al., 2020). The ambulatory ECG devices have been sufficiently miniaturized, wearable and connected to high-speed mobile networks with the promise to give patients high-quality yet affordable health monitoring services at home. To enable the adoption of remote ECG monitoring services in the general population, reliable automatic ECG analysis and diagnosis technology is necessary as analyzing the vast amount of monitoring data is far beyond the capability of human physicians. Although the technologies for automatic ECG analysis have been developed for decades, current technologies cannot replace human physicians for diagnosis because they have limited generalization ability to cope with the diverse artifacts and inter-patient variations in the ECG signals (Alday et al., 2020; Siontis et al., 2021). Therefore, novel technologies for generalizable detection of arrhythmias are in urgent demand.

Beat-by-beat arrhythmia detection, determining the rhythm type of each heartbeat in an ECG recording, is essential for the analysis of ambulatory ECG. According to the ANSI/AAMI EC57:2012 standard (AAMI, 2012), a beat-by-beat arrhythmia detection software should discriminate five types of heartbeats: ventricular ectopic beat (VEB or V), supraventricular ectopic beat (SVEB or S), ventricular fusion beat (F), ambiguous beat (Q), and beat of all other types (N). In particular, the detection performances of SVEB and VEB are of major interest to health care practitioners and constitute the evaluation metrics for the detectors as recommended by the ANSI/AAMI standard. An accurate beat-by-beat arrhythmia detector has several important implications for clinical practices. The detected beat-wise rhythm types manifest the occurrence time of each arrhythmia episode which is necessary evidence to correlate the detected arrhythmias with the symptoms recorded by the patients. Besides, the detected types of each heartbeat in the recording can be used to measure the burdens (i.e., the proportions in all heartbeats) of VEB and SVEB which are important indicators in evaluating cardiac function (Baman et al., 2010), assessing the effectiveness of treatments (Deyell et al., 2012), and predicting the risk of malignant diseases, such as stroke, heart failure and sudden death (Binici, 2010; Marcus, 2020). Furthermore, the fine-grained beat-wise rhythm types can be further used to identify some complex patterns of arrhythmias, such as ventricular/supraventricular tachycardia, bigeminy, and trigeminy.

**FIGURE 1:**
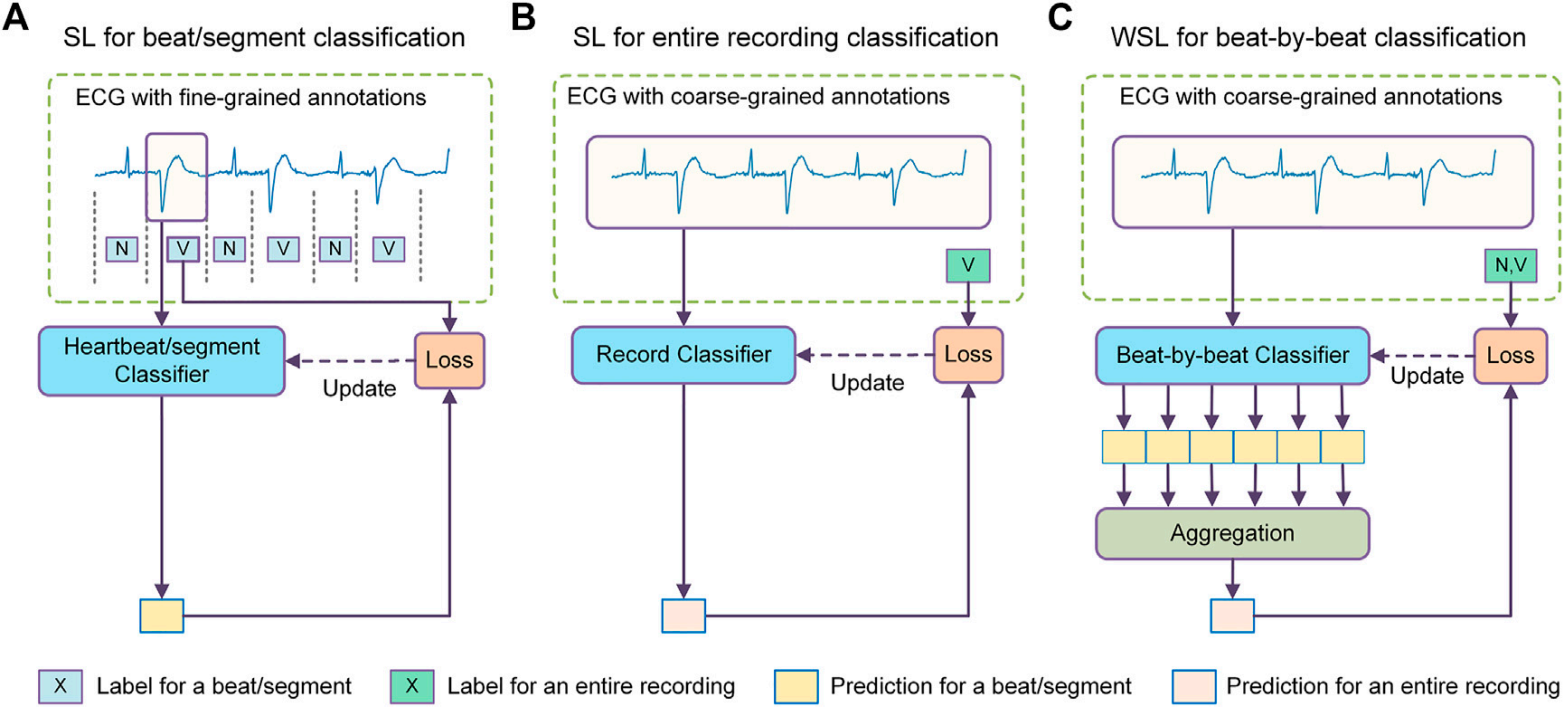
Comparison between different methodologies of machine learning for arrhythmia detection. **(A)** The supervised learning (SL) method for heartbeat/segment classification which uses fine-grained annotations for model training and achieves fine-grained predictions. **(B)** The SL method for ECG recording classification which uses coarse-grained labels as supervision and achieves coarse-grained predictions. **(C)** The weakly supervised learning (WSL) method for beat-by-beat classification which uses coarse recording labels for model training but achieves fine-grained predictions. N = normal or bundle branch block beat. SVEB = supraventricular ectopic beat. VEB = ventricular ectopic beat.

The state-of-the-art methods for beat-by-beat arrhythmia detection are generally based on machine learning (ML), a methodology to guide the models to learn detection rules from a training dataset. Typically, the ECG signal is segmented into individual heartbeats, each of which is then fed into a classifier to determine its rhythm type, as shown in Figure 1A. The classifier is usually trained in a supervised learning methodology, where each training beat is annotated with a corresponding rhythm type. According to the split of training and test sets, the classifiers can be further categorized into two types: intra-patient classifier, of which the training and test data are from the same group of patients (Kiranyaz et al., 2016; Li et al., 2018; Degirmenci et al., 2021), and inter-patient classifier, of which the training and test data are from non-overlapping patient populations (Raj and Ray, 2018; Guo et al., 2019; Niu et al., 2020). The intra-patient classifiers are suitable to develop personalized arrhythmia detectors, while the inter-patient classifiers aim to provide diagnostic models for general populations. Intra-patient classifiers usually perform much better than inter-patient classifiers because they have been fine-tuned for the target population. This in turn suggests that it is more challenging to develop models with good generalization abilities to deal with data from unseen patients. In real-world medical settings, the ECGs are obtained from large, diverse populations, which makes the classifier’s generalization ability of critical importance.

A key reason for the poor generalization ability of current methods is that the training data come from a small population. For example, the MIT-BIH Arrhythmia Database, a dataset used by dozens of studies for classifier training, contains only 48 ECG recordings collected from 47 subjects (Moody and Mark, 2001). Since the training data only reflect the characteristics of a few people, it is naturally difficult for the model learner to learn the discrimination features applicable to data from general population. For example, it has been demonstrated that patients with COVID-19 exhibit obvious ECG changes, based on which an automatic diagnostic model for COVID-19 has been developed and achieved a quite high accuracy and F1 score (both ≥93.0%) (Ozdemir et al., 2021). These COVID-19-related ECG changes represent a new class of conditions that may not be considered by previous arrhythmia detectors and may affect the generalization ability of those detectors. Besides, the models tend to over-fit the small amount of training data and thus impair their generalization ability. The small sample sizes of the training datasets are partly because of the high cost of the beat-by-beat annotation work. In order to ensure the correctness of the labels, independent labeling by multiple experts is often required and the disputed samples should resort to an expert committee for adjudication. Another challenge of machine learning for arrhythmia detection is class imbalance, where samples of normal sinus rhythm tend to predominate in the datasets. Some strategies have been proposed to address this problem. For example, Lu *et al.* utilized focal loss to address the class imbalance in ECG classification (Lu et al., 2021).

In view of the limitation of annotated training data, some researchers have explored new methods to improve the generalization ability of ECG classifiers, such as unsupervised learning and semi-supervised learning. The unsupervised learning methods have been developed to mine unlabeled ECG data for representative learning (Rajan and Thiagarajan, 2018), domain adaptation (Li et al., 2021; Wang et al., 2021), and data augmentation (Golany and Radinsky, 2019; Wulan et al., 2020). Some semi-supervised learning methods that use both labeled and unlabeled data for model training have also been developed to fine-tune the classifier for the target patient without the need for patient-specific labeled data (Zhai et al., 2020). Although these methods contribute to the improvement of generalization ability, the limited labeled data still plays a very central role in the training of these models and induce a high risk of overfitting.

This study explores the possibility of another learning approach, i.e., weakly supervised learning (WSL), to improve the generalization ability of beat-by-beat arrhythmia detectors. Unlike previous methods that utilize unlabeled samples or synthesize new samples, the WSL approach tries to train a model with incomplete, inexact, or inaccurate annotations that are usually much easier to obtain (Zhou, 2018). In the domain of ECG classification, there are a large amount of ECG data annotated with coarse-grained labels, i.e., a recording (typically several to tens of seconds) is labeled as a whole (Liu et al., 2018; Alday et al., 2020). Since the rhythm types of individual heartbeats in these datasets are not annotated, these datasets are mainly used to train recording classifiers that determine if certain anomalies are present in an ECG recording, as shown in Figure 1B, in previous studies. Nevertheless, the recording labels indicate the rhythm types of an unknown subset of heartbeats in the recording, which can provide a form of weak supervision for the model training. In addition, as these datasets reflect diverse signal artifacts and inter-patient variations, they may help prevent overfitting and improve the generalization ability of the heartbeat classifiers.

Several issues need to be addressed for applying WSL to beat-by-beat arrhythmia detection. Firstly, what is the mapping relationship between the target labels of individual heartbeats and the labels of their recording? For this study, the recording labels in datasets such as the PhysioNet/CinC Challenge 2020 datasets clearly reflect the presence of SVEB or VEB in the recordings, which is critical to a successful application of WSL. Secondly, how to construct a beat-by-beat classifier that can be trained under the supervision of recording labels? As the true heartbeat labels are not available, a mechanism is needed to guide the optimization of the heartbeat classifier based on the true recording labels and the mapping relationships between heartbeat labels and recording labels. Finally, how to address the ill-posed problem that the constraints of recording labels can be satisfied by different hypotheses of the heartbeat rhythms? The ill-posed problem usually arises when two types of samples always occur concomitantly (Choe et al., 2020). For example, SVEBs and sinus beats usually occur alternately and have similar waveforms, which may confuse the classifier in discriminating these two kinds of beats since swapping their categories can also map to the same recording labels. Therefore, the ill-posed problem must be addressed to ensure the stability of the heartbeat classifier.

In this study, we propose a deep-learning-based WSL framework for beat-by-beat arrhythmia detection (WSDL-AD), as shown in Figure 1C, which can be trained with just coarse record-level labels in an end-to-end manner. In this framework, the model first makes local predictions for each heartbeat, and then maps the heartbeat predictions to the prediction of the recording labels by an aggregation mechanism. The model can be optimized by gradient descent, where the gradients of the recording predictions are back-propagated through the aggregation layer to calculate the gradients of the heartbeat predictions. Thus, the heartbeat classifier can be optimized according to the coarse recording labels. To address the ill-posed problem, we design a two-stage training strategy: a supervised pre-training stage with small amounts of heartbeat labels, and a weakly-supervised training stage with large amounts of recording labels. In addition**,** we introduce some techniques into the WSDL-AD framework to enhance the model performance. 1) To assist the model in utilizing contextual information, two knowledge-based features, namely relative RR interval and RR entropy, are proposed. 2) Considering the heartbeats vary in length and are unevenly distributed over time, we propose a masked aggregation mechanism that can select a representative prediction for each heartbeat for aggregation without the need to split the ECG signal into heartbeat segments.

The remainder of this paper is organized as follows. In section 2, the proposed WSDL-AD framework and the datasets for model training and evaluation are described in detail. The experimental setup and results are present in section 3. Section 4 compares our results with that of other studies and discusses the implications and limitations of this work. Finally, we conclude this work in section 5.

## 2 Materials and Methods

### 2.1 Datasets

We use multiple coarsely annotated and finely-annotated datasets in this study. The coarsely-annotated datasets are from the PhysioNet/CinC challenge 2020/2021 (Alday et al., 2020; Reyna et al., 2021), including two China physiological signal challenge datasets (CPSC and CPSC-Extra) (Liu et al., 2018), the Physikalisch Technische Bundesanstalt extension (PTB-XL) dataset (Wagner et al., 2020), the Georgia 12-lead ECG challenge dataset (G12EC), and the Chapman University, Shaoxing People’s Hospital and Ningbo First Hospital database (Chapman-Shaoxing-Ningbo) (Zheng et al., 2020). There are a total of 87,653 12-lead ECG records in these datasets. Each record was annotated as a whole using the SNOMED CT codes (Donnelly, 2006). The ANSI/AAMI standard recommends five classes for the arrhythmia detector, namely VEB, SVEB, F, Q, and N (AAMI, 2012). In addition, as recommended by the standard, a detector is neither penalized nor rewarded for its treatment of F and Q. Therefore, in this work, we only deal with the classification of VEB, SVEB, and N. The data of F and Q are excluded from the evaluation. The mapping between the original labels and the ANSI/AAMI classes is available in Table 1. Statistics of the datasets are shown in Table 2.

**TABLE 1:** The mapping between the dataset labels and the classes suggested by the ANSI/AAMI standard. The dataset labels are in the parentheses following their corresponding class names.

ANSI/AAMI	SNOMED CT codes	PhysioBank labels
N	All labels except that mapped to SVEB, VEB	Normal beat (N)
		Left bundle branch block beat (L)
		Right bundle branch block beat (R)
		Atrial escape beat (e)
		Nodal (junctional) escape beat (j)
SVEB	Premature atrial contraction (284470004)	Atrial premature beat (A)
	Supraventricular premature beats (63593006)	Aberrated atrial premature beat (a)
		Nodal (junctional) premature beat (J)
		Supraventricular premature or ectopic beat (S)
VEB	Premature ventricular contractions (427172004)	Premature ventricular contraction (V)
	Ventricular premature beats (17338001)	Ventricular escape beat (E)
	Ventricular ectopic beats (164884008)	

**TABLE 2:** The compositions of the datasets.

Datasets	Recording numbers	Recording lengths	Sampling rate (Hz)	Annotations			Annotation unit
				N	VEB	SVEB	
CPSC	6,877	6–144 s	500	5,564	700	616	record
CPSC-Extra	3,453	8–98 s	500	3,150	194	124	record
PTB-XL	21,837	10 s	500	20,194	1,154	555	record
G12EC	10,334	5–10 s	500	9,336	395	640	record
Chapman-Shaoxing-Ningbo	45,152	10 s	500	42,536	1,385	1,321	record
MITBIH-AR-DS1	22	30 min	360	45,869	3,789	945	beat
MITBIH-AR-DS2	22	30 min	360	44,264	3,221	1837	beat
MITBIH-SUP	78	30 min	128	162,368	9,950	12,207	beat
INCART	75	30 min	257	153,673	20,012	1960	beat

CPSC, the China physiological signal challenge; PTB-XL, the Physikalisch Technische Bundesanstalt extension dataset; G12EC, the Georgia 12-lead ECG challenge dataset; MITBIH-AR-DS1, the DS1 of MIT-BIH arrhythmia database; MITBIH-AR-DS2, the DS2 of MIT-BIH arrhythmia database; MITBIH-SUP, the MIT-BIH supraventricular arrhythmia database; INCART, the St. Petersburg INCART arrhythmia database; N, normal or bundle branch block beat; SVEB, supraventricular ectopic beat; VEB, ventricular ectopic beat.

The finely annotated datasets used in this study include the MIT-BIH arrhythmia database (MITBIH-AR) (Moody and Mark, 2001), the MIT-BIH supraventricular arrhythmia database (MITBIH-SUP) (Greenwald et al., 1990), and the St. Petersburg INCART arrhythmia database (INCART) (Goldberger et al., 2000). The MITBIH-AR dataset is further divided into two subsets as in (De Chazal et al., 2004), namely MITBIH-AR-DS1^1^ (or DS1) and MITBIH-AR-DS2^2^ (or DS2), which contain ECG records from non-overlapping patient groups and have similar category distributions. This division is widely used in previous studies (Mar et al., 2011; Raj and Ray, 2018; Niu et al., 2020), where the models are trained on DS1, and tested on DS2. For comparison purposes, we also adopt this division in this study. All these datasets contain two-lead ECG signals with physician-reviewed beat-by-beat annotations in the PhysioBank labels, which can be mapped to the AAMI classes as in Table 1. The compositions of the datasets are shown in Table 2.

In this study, the signal in the lead II of each recording is used for beat-by-beat arrhythmia detection. For recordings in MITBIH-AR, the modified lead II (MLII) is used instead. If the lead configurations are unavailable, such as in MITBIH-SUP, the signal in the first lead is used.

### 2.2 Overview of the WSDL-AD Framework

**FIGURE 2:**
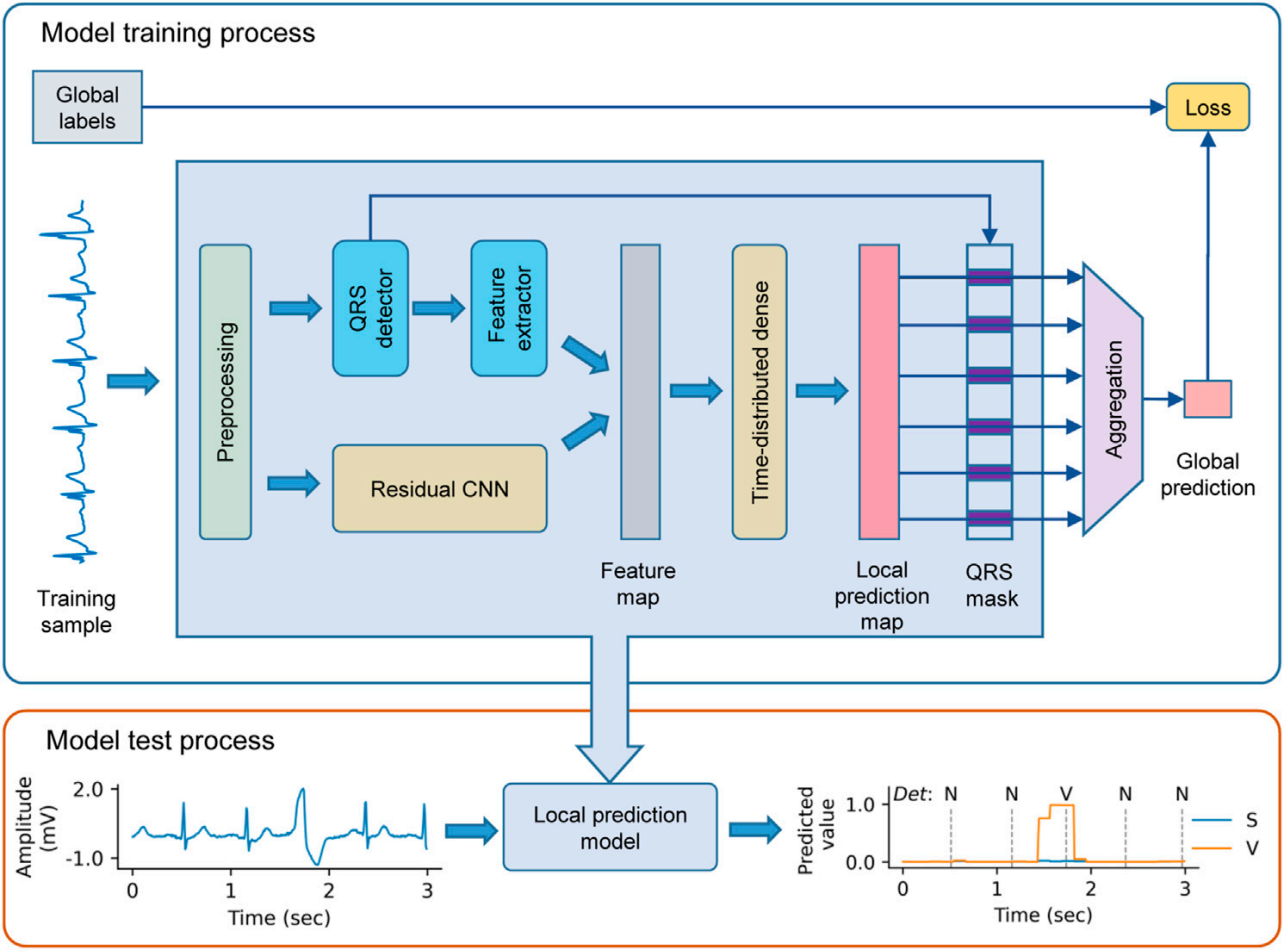
The schematic diagram of the weakly supervised deep learning framework for arrhythmia detection (WSDL-AD). CNN = convolutional neural network. N = normal or bundle branch block beat. S = supraventricular ectopic beat. V = ventricular ectopic beat.

We propose the WSDL-AD framework for beat-by-beat arrhythmia detection, as shown in Figure 2. The framework is input with an ECG signal of variable length. The input signal is first preprocessed to unify the signal configurations (such as sampling rate and amplitude) and eliminate noise. Then, feature maps are extracted from the signal by a residual convolutional neural network (ResNet) and domain-knowledge-based methods respectively. Based on the features, the framework makes local predictions in the granularity of a sampling point. Then the predicted rhythm for each heartbeat is obtained by selecting the prediction at its R peak. Finally, the beat-level predictions are aggregated into the global prediction, whereby the loss value for the prediction can be calculated according to the global annotations to enable the model to be trained end-to-end.

### 2.3 Preprocessing

The preprocessing is mainly aimed to eliminate the noise and unify the sampling rate and amplitude of the ECG signals. Each ECG recording is first processed by a moving average filter (the window size is one second) to estimate the baseline wander which mainly originated from the offset and low-frequency noises in the signal. The estimated baseline wander is then removed by subtracting it from the signal. The signal is also processed by a band-pass filter (0.1–30 Hz) to suppress noises in other bands. In addition, we resample the signal to 125 Hz and normalize the signal to have mean zero and variance one. Among these datasets, the MITBIH-SUP has a frequency (128 Hz) very similar to our target frequency, so signals of this dataset are not resampled. But the frequencies of other datasets are all much higher than the target frequency, so signals in these datasets are downsampled to 125 Hz.

For the coarsely-annotated training data, a tricky situation is that if a recording contains both sinus and arrhythmia episodes, its annotation usually does not include the label for sinus rhythm. To address this problem, we complement the labels based on the rules of co-occurrence of different rhythms. Specifically, if the label set of a recording contains SVEB or VEB, and contains neither supraventricular tachyarrhythmia nor idioventricular rhythm, it is very likely that beats of N are also present in the recording. So, in this case, we add the label N to the label set of the recording for complementing.

### 2.4 Knowledge-Based Features

Contextual information, e.g., the variation of RR intervals, is essential for the detection of many arrhythmias. In order to facilitate the utilization of contextual information in arrhythmia detection, we design two context-relative features based on domain knowledge. The extraction of the two features requires the positions of QRS complexes, which are usually not directly available, and need to be detected by some algorithms. In this study, the QRS complexes are detected by a U-net-based model that has been proposed in a previous study (He et al., 2020).

#### 2.4.1 Relative RR Interval

The change of current RR interval relative to the contextual normal sinus RR intervals supplies important information for the detection of many arrhythmias including SVEB and VEB. So, we design a feature, named relative RR interval, to represent this information: $I_{R}=\left(I_{N}-I_{A}\right)/I_{N}\cdot s$ , where $I_{R}$ is the relative RR interval, $I_{A}$ is the absolute RR interval, $I_{N}$ is the representative normal RR interval in the context, and *s* is a scaling parameter. Here, we use the mean RR interval of the context to approximate $I_{N}$ for simplicity of calculation: $I_{N}\approx mean\left(I\right)$ , where I is the set of RR intervals in the context. Note that $I_{R}$ is negatively related with $I_{A}$ . $I_{R}$ is positive when $I_{A}<I_{N}$ , while $I_{R}$ is negative when $I_{A}>I_{N}$ . Due to the wide adoption of rectified linear unit (ReLU), many DNN models are more sensitive to positive values. Therefore, our design can induce the DNN to be more sensitive to the shortening of RR interval which is an important indicator of ectopic heartbeats. Besides, $I_{R}$ is normalized by $I_{N}$ in the formula to better reflect the degree of relative changes.

In selecting the length of the context for estimating $I_{N}$ , a trade-off should be considered between the accuracy and robustness: the longer the context is, the more robust the mean RR interval is to local disturbances, but the less accurately it reflects a temporal fluctuation of the sinus RR interval. In this work, we set the context length to 60 RR intervals with the current RR interval at the middle of the context. And when the number of RR intervals in a recording is less than 60, all the RR intervals are used as the context. The scaling parameter, *s*, is used to increase the feature’s contribution in the prediction since the raw value is usually small and easy to be ignored by the classifier. In our implementation, *s* is set to 10.

The relative RR intervals of a recording are organized in a feature map, which has the same length as the ECG. In this way, the feature map can be easily combined with the DNN-extracted feature maps, and used in the local predictions. The feature map is organized as follows: the points in the region of a heartbeat are assigned the feature value of the corresponding heartbeat. Here, we define the region of a heartbeat as the portion between the midpoint of its preceding RR interval and the midpoint of its succeeding RR interval.

#### 2.4.2 RR Entropy

The regularity and stationarity of the RR intervals in the context also provide important diagnostic information. We measure this information by another feature, named RR entropy. The RR entropy is calculated by the sample entropy (SampEn) method (Richman and Moorman, 2000). SampEn is the negative natural logarithm of the empirical probability that two templates (i.e., segments) of length *m*+1 from the input sequence match each other given that their sub-templates containing the corresponding first *m* sampling points match. In our implementation, *m* is set to 1, and the threshold to determine whether two templates match is set to 0.05. Before the entropy calculation, the RR intervals are divided by their median value for normalization. Since the entropy can fluctuate with time, we calculate its values dynamically in a sliding window. Similar to the selection of the context length for estimating normal RR interval, the selection of window size for entropy calculation should also take into account the balance between accuracy and robustness. The window size is set also to 60 RR intervals in our implementation. And the method for window selection is the same as that for specifying the context in normal RR interval estimation. The calculated RR entropies are also organized in a feature map, where the result of each moving window calculation is mapped to the region of the central heartbeat (typically the 30th heartbeat) of the window. Any sampling point that is not mapped in the above process is assigned the value of its neatest mapped neighbor.

**FIGURE 3:**
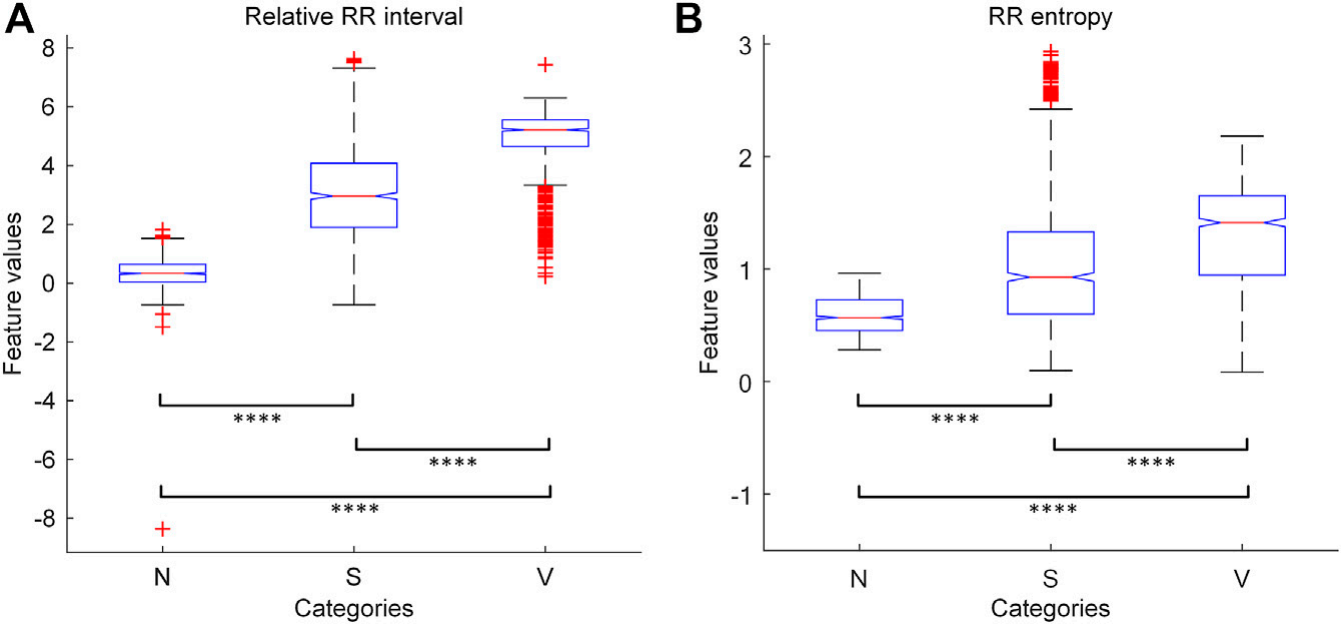
The results of one-way ANOVA with multiple pairwise comparison for the knowledge-based features. **(A)** The results for relative RR interval. **(B)** The results for RR entropy. The symbol “****” indicates that the corresponding two groups are significantly (*p* < 0.0001) different from each other in features values. N = normal or bundle branch block beat. S = supraventricular ectopic beat. V = ventricular ectopic beat.

To assess the discriminative abilities of the features, we randomly sample 2,700 heartbeats from the MITBIH-AR-DS1 (900 for each category), and apply one-way ANOVA test on their feature values grouped by categories. The results show that the values of both features are significantly different (*p* < 0.0001) among these categories. We also perform multiple pairwise comparisons on the feature values, and the results are given in Figure 3. We found that the feature values are significantly different (*p* < 0.0001) between each pair of categories. Therefore, these knowledge-based features have certain discernibility for categories and will contribute to the classification task.

### 2.5 DNN-Based Feature Extraction and Local Prediction

**FIGURE 4:**
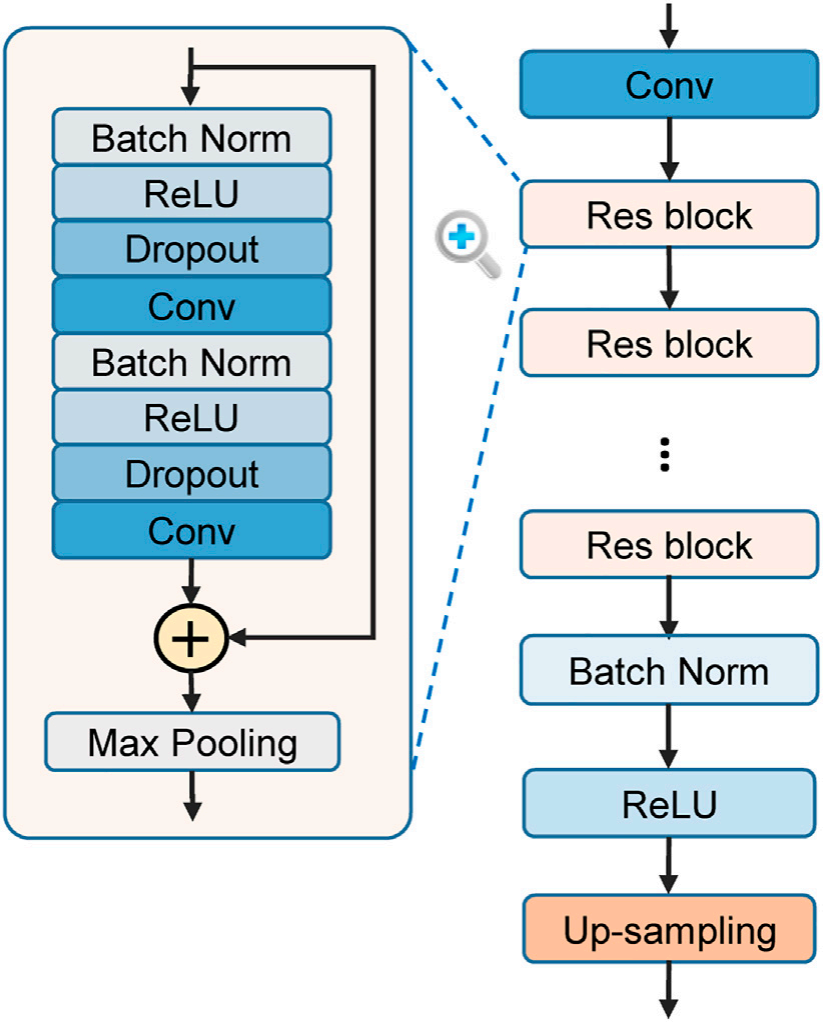
The structure of residual convolutional network (ResNet) for feature learning. Res block = residual block. Conv = convolutional layer. Batch Norm = batch normalization. ReLU = rectified linear unit.

The ResNet is used to automatically extract features from the ECG. It consists of a stack of residual convolutional blocks (Res blocks) as shown in Figure 4. Each Res block contains two convolutional (Conv) layers and some assistant layers, including batch normalization (BN) (Ioffe and Szegedy, 2015), rectified linear unit (ReLU) (Nair and Hinton, 2010), and Dropout (Srivastava et al., 2014). The output of a block’s last convolutional layer is merged with the block’s input by element-wise addition, as suggested by the original ResNet (He et al., 2016). A max-pooling layer with a pool size of two then compresses the merged output to half of its original length. The blocks are connected in series, with the output of the previous one serving as the input of the latter. The outputs of the last block are a series of feature maps characterizing each temporal slice of the input recording. To align with the knowledge-based features, these DNN-extracted feature maps are up-sampled along the time dimension to the same length of the input. Then, these feature maps are concatenated with the feature maps of knowledge-based features along the feature dimension, and jointly used for the local prediction. This architecture contains several hyperparameters which may affect the model performance. We optimize the hyperparameters by grid search: different choices of residual blocks number (3, 4, 5, and 6), convolutional kernel number (16, 32, and 64), convolutional kernel length (8, 16, and 32), and dropout rate (0, 0.25, and 0.5) are tested to find the combination that achieves the best performance on the validation set (MITBIH-AR-DS1). After the hyperparameters optimization, the ResNet consists of 4 Res blocks. In each Res block, each convolutional layer contains 32 kernels with a kernel length of 8. The parameters of each convolutional layer are initialized by the method proposed in (He et al., 2015), which takes the ReLU into account and allows for very deep models. The dropout rate of each dropout layer is 0.25.

Based on the feature map merged from DNN features and knowledge-based features, the local predictions are made by a time distributed dense (TDD) layer, which leverages a dense layer (i.e., fully-connected layer) to process each temporal element separately. The cell number of the TDD layer is equal to the number of considered rhythm types. The output of the TDD layer at each temporal element is then processed by the softmax function to calculate probability of each considered rhythm occurring at the slice.

### 2.6 Aggregation for Global Prediction

The aggregation mechanism, mapping the local predictions to the global prediction, is a critical part in the WSDL-AD framework. Here, we introduce the method of applying the traditional aggregation mechanisms to this framework, and also propose the masked aggregation mechanism.

#### 2.6.1 Traditional Aggregation Methods

A series of aggregation methods have been proposed in previous studies for computer vision, including global average pooling (GAP) (Zhou et al., 2016), global maximum pooling (GMP) (Pathak et al., 2014), and Log-Sum-Exp (LSE) (Pinheiro and Collobert, 2015). Here, we formalize these methods for application to the mapping between the local predictions and the global prediction. GAP averages all local predictions to obtain the global prediction: $GAP{\left(\hat{Y}\right)}_{c}=\frac{1}{n}\overset{n}{\underset{i=1}{\sum }}{\hat{y}}_{i,\;c}$ , where $\hat{Y}$ is the collection of local predictions, ${\hat{y}}_{i,\;c}$ is the prediction for rhythm *c* at the *i*th sampling point. We argue that GAP is not suitable for use in the WSDL-AD framework, because some arrhythmias, e.g., ectopic beats, may account for only a very small fraction of a recording, and the predictions for them will be ignored by GAP. In contrast, GMP selects the maximum local prediction for a class as its global prediction: $GMP{\left(\hat{Y}\right)}_{c}=\underset{i\in \left\{1,\;\ldots ,\;n\right\}}{\max}{\hat{y}}_{i,\;c}$ . By using GMP, a rhythm is considered to be present in a recording as long as it occurs at some time in the recording regardless of the duration. One possible problem of GMP is that it may underestimate the true region of an object (Kolesnikov and Lampert, 2016), because the gradient of the prediction loss will be saturated (extremely close to 0) as long as the maximum activation for the existing arrhythmia is extremely close to 1. One solution for this problem is to make a compromise between GAP and GMP. For example, LSE is a convex approximation of the max function (Pinheiro and Collobert, 2015): $LSE{\left(\hat{Y}\right)}_{c}=\left(1/b\right)\text{log}\left(\;\left(1/n\right)\overset{n}{\underset{i=1}{\sum }}\text{exp}\left(b{\hat{y}}_{i,\;c}\right)\right)$ , where *b* > 0 is a hyper-parameter that controls the degree of the approximation to GMP. Increasing *b* will push the function closer to GMP, while decreasing *b* will make the function closer to GAP.

However, there are some problems when directly applying these aggregation methods to the local predictions. Firstly, the local predictions are corresponding to sampling points rather than heartbeats. Although we can divide these local features and predictions into individual heartbeats, the variable length and uneven distribution of heartbeats can make an accurate division difficult and increase the complexity of the model structure. Secondly, due to the significant morphological differences between the subwaves (e.g., P wave, QRS complex, and T wave) of a heartbeat, the local predictions at different parts of a heartbeat may be inconsistent, i.e., different parts of a heartbeat are classified to different classes. These inconsistent predictions are unreasonable and will increase the difficulty of model optimization. To address these problems, we propose a masked aggregation mechanism.

#### 2.6.2 Masked Aggregation

The inconsistent predictions within a heartbeat are mainly due to the morphological differences between the subwaves (e.g., P, QRS, and T) of a heartbeat. This problem can be circumvented by selecting the prediction at a certain point in a heartbeat (i.e., the reference point) as the representative prediction for the beat. To ensure the representativeness of the selected predictions, we propose to aggregate only the selected predictions to obtain the global prediction. In this way, only the selected predictions are optimized according to the gradient of the global prediction loss, and thus they will be representative of the beats after the training. Since other predictions are masked out in the aggregation, we call this mechanism masked aggregation. In this work, we choose the R peak as the reference point of a heartbeat because it can be usually accurately recognized by certain algorithms (He et al., 2020). On the selected local predictions, the aggregation methods mentioned above can also be applied. Here, we combine masked aggregation with GMP to get masked global max pooling (MGMP): $MGMP{\left(\hat{Y}\right)}_{c}=\underset{i\in R}{\max}{\hat{y}}_{i,\;c}$ , where *R* denotes the set of reference points. By selecting only the local predictions at the reference points, the space of possible solutions for local predictions will be significantly reduced, because the reference points in a recording are a few orders of magnitude less than the sampling points. Furthermore, the masked aggregation induces the model to learn features around the reference points so that the learned features at different reference points are semantically comparable between each other.

### 2.7 Loss Calculation

The global predictions are used for the loss calculation since only record-level labels are available in our WSL setting. The loss function should support multi-label classifications, because multiple rhythms may coexist in a single ECG record. Here, we use the binary cross-entropy as the loss function for the training of our models:

$$L_{\theta }\left(X,T\right)=-\frac{1}{\left|C\right|}\overset{\left|C\right|}{\underset{c=1}{\sum }}\left(t_{c}log\left(f{\left(X;\theta \right)}_{c}\right)+\left(1-t_{c}\right)log\left(1-f{\left(X;\theta \right)}_{c}\right)\right)\;$$ (1)

where *X* is a recording of the training set, *T* is the record-level label set of *X*, f is the prediction model, and |C| is the number of considered classes. $t_{c}$ is an indicator of the presence of class *c* in the label set *T*: if $c\in T,\;t_{c}=1$ ; otherwise, $t_{c}=0$ . Besides, because N is much more common than SVEB and VEB in clinic, there is an extreme imbalance between these classes. For this problem, we assign different weights to the training samples of different classes in the loss calculation:

$$L_{\theta }\left(D\right)=\frac{1}{M}\underset{\left(X_{i},T_{i}\right)\in D}{\sum }w_{\left(X_{i},T_{i}\right)}L_{\theta }\left(X_{i},T_{i}\right)$$ (2)

where $L_{\theta }\left(D\right)$ is the loss for the training set *D*, $w_{\left(X_{i},T_{i}\right)}$ is the weight for the sample $\left(X_{i},T_{i}\right)$ , and *M* is the number of samples in the dataset. In our implementation, after tuning the weight parameters with experiments, the weight for a sample with SVEB or VEB is set to 2, the weight for a sample with both SVEB and VEB is set to 4, while the weight for a sample with neither SVEB nor VEB is set to 0.1.

### 2.8 Two-Stage Training Strategy

To address the ill-posed problem of WSL, we propose a two-stage training strategy. In the first stage, the model is pre-trained in SL with small amounts of samples with heartbeat labels. Then, in the second stage, the pre-trained model is further trained in WSL with large amounts of coarsely-labeled ECG data. The SL-based pre-training is implemented by omitting the aggregation part of the WSDL-AD framework and applying the supervision directly to the local predictions. The loss value is calculated using categorical cross-entropy on the predictions at the R peaks, as in (3).

$$L_{\theta }\left(X,Y\right)=-\frac{1}{\left|R\right|}\underset{i\in R}{\sum }\overset{\left|C\right|}{\underset{c=1}{\sum }}\left(y_{i,c}\log\left(g{\left(X;\theta \right)}_{i,c}\right)\right)$$ (3)

where  g is the local prediction model, R denotes the set of heartbeat positions, Y denotes the set of heartbeat labels. $y_{i,c}$ is an element of Y : if the heartbeat at the *i*th sampling point belongs to category $c,\;y_{i,c}=1$ ; otherwise, $y_{i,c}=0$ . The idea behind this strategy is that the SL-based pre-training can initialize the model with proper parameters that may prevent the subsequent WSL-based training from going in the wrong direction.

## 3 Results

In this section, we describe the experimental setup and results to evaluate the performance of the WSDL-AD framework in beat-by-beat arrhythmia detection. Ablation studies are also conducted to assess the influence of our proposed techniques on the model’s performance.

### 3.1 Experimental Setup

For comparison between different ML methodologies, we conduct the experiments in three settings: 1) SL setting, where the model is trained with full supervision; 2) WSL setting, where the model is trained with only weak supervision; and 3) the SL + WSL setting, where the model is pre-trained with full supervision on a small dataset, and then trained with weak supervision on a large dataset. Details of each setting are shown in the following subsections.

We implement the models based on the Tensorflow and train the models on a workstation with a CPU running at 3.5 GHz, an NVIDIA Quadro k6000 GPU, and 64 GB of memory. The method used for model optimization is Adaptive Moment Estimation (Adam) (Kingma and Ba, 2014), where *β*_1_ is 0.9, *β*_2_ is 0.999, and the learning rate is 0.001. The training process is terminated when the mean F1 score for all categories on the validation set doesn’t increase over 10 epochs. The source code is available at https://github.com/sdnjly/WSDL-AD.

By comparing the model predictions with the annotations, we calculate several metrics to evaluate the model performance. These metrics include sensitivity (*Sen*), specificity (*Spe*), positive predictivity (*Ppr*), accuracy (*Acc*), *F*_1_ score, and average precision (*AP*). Formulas for calculating these metrics are as follows:

$$Sen=\;\frac{TP}{TP+FN}$$ (4)

$$Spe=\;\frac{TN}{FP+TN}$$ (5)

$$Ppr=\;\frac{TP}{TP+FP}$$ (6)

$$Acc=\;\frac{TP+TN}{TP+FP+TN+FN}$$ (7)

$$F_{1}=\;\frac{2\times Sen\times Ppr}{Sen+Ppr}$$ (8)

$$AP=\underset{n}{\sum }\left(Sen_{n}-Sen_{n-1}\right)Ppr_{n}$$ (9)

where *TP* denotes true positive predictions, *TN* denotes true negative predictions, *FP* denotes false positive predictions, and *FN* denotes false negatives predictions. $Sen_{n}$ and $Ppr_{n}$ are the sensitivity and positive predictivity at the *n*th threshold of the precision-recall curve (PRC) (Chen, 2003).

### 3.2 The SL Setting

For training the SL model, recordings of MITBIH-AR-DS1 are split into segments of 20 s 80% of the segments are randomly selected as the training set, and the remaining 20% are used as the validation set. The validation set is just used for hyperparameters tuning and early stopping of the training process. The trained model is tested on the other three completely independent and finely annotated datasets, including MITBIH-AR-DS2, MITBIH-SUP, and INCART. The metric scores on these test sets are used for the final evaluation of the model performance.

The test results on each dataset and the total test data are shown in Table 3. For the detection of SVEB, the SL model achieves high scores in *Spe* and *Acc*, but has very low scores in *Sen*, *Ppr*, and *F*_1_. For example, the *Spe* scores of the SL model on MITBIH-AR-DS2, MITBIH-SUP, and INCART are 0.994, 0.981, and 0.993, respectively, whereas its *Sen* scores on these datasets are only 0.066, 0.130, and 0.590, respectively. The high scores of *Spe* and *Acc* can be attributed to the extreme class imbalance of the test sets, where only a tiny minority of the samples belong to SVEB. And the low scores of *Sen*, *Ppr*, and *F*_1_ are a true reflection of the poor ability of the SL model in detecting SVEB. The test scores for VEB detection are much higher than those for SVEB detection. Besides, the model performances are different from dataset to dataset. For example, the scores of *Ppr* and *F*_1_ for VEB detection on the MITBIH-SUP dataset are much lower than that on the other datasets. These differences may result from the diversity of data distribution among these test sets.

**TABLE 3:** Experimental results of different training setting on the evaluation datasets.

Test set	Experimental setting	N					S					V				
		*Sen*	*Ppr*	*Spe*	*Acc*	*F*_*1*_	*Sen*	*Ppr*	*Spe*	*Acc*	*F*_*1*_	*Sen*	*Ppr*	*Spe*	*Acc*	*F*_*1*_
MITBIH-AR-DS2	SL	0.985	0.959	0.634	0.949	0.972	0.066	0.286	0.994	0.959	0.108	0.935	0.873	0.991	0.987	0.903
	WSL	0.990	0.987	0.883	0.979	0.988	0.806	0.799	0.992	0.985	0.803	0.902	0.950	0.997	0.990	0.925
	SL + WSL	0.990	0.992	0.928	0.983	0.991	0.886	0.785	0.991	0.987	0.832	0.916	0.956	0.997	0.992	0.936
MITBIH-SUP	SL	0.932	0.941	0.574	0.889	0.936	0.130	0.323	0.981	0.924	0.186	0.826	0.434	0.939	0.933	0.569
	WSL	0.991	0.951	0.629	0.947	0.971	0.325	0.776	0.993	0.949	0.458	0.803	0.774	0.987	0.977	0.788
	SL + WSL	0.986	0.962	0.717	0.954	0.974	0.452	0.705	0.987	0.951	0.551	0.795	0.768	0.986	0.976	0.782
INCART	SL	0.991	0.978	0.844	0.973	0.985	0.590	0.487	0.993	0.989	0.534	0.826	0.945	0.994	0.975	0.882
	WSL	0.990	0.988	0.914	0.981	0.989	0.852	0.575	0.993	0.991	0.687	0.886	0.950	0.994	0.982	0.917
	SL + WSL	0.993	0.990	0.933	0.986	0.992	0.927	0.519	0.990	0.990	0.665	0.880	0.976	0.997	0.984	0.926
The Total Test Set	SL	0.964	0.959	0.701	0.932	0.961	0.179	0.371	0.988	0.956	0.242	0.837	0.696	0.968	0.957	0.760
	WSL	0.990	0.971	0.782	0.965	0.981	0.445	0.721	0.993	0.972	0.550	0.862	0.893	0.991	0.980	0.878
	SL + WSL	0.990	0.978	0.835	0.971	0.984	0.560	0.669	0.989	0.972	0.610	0.858	0.906	0.992	0.981	0.882

N, normal or bundle branch block beat; S, supraventricular ectopic beat; V, ventricular ectopic beat; SL, supervised learning; WSL, weakly supervised learning; Sen, sensitivity; Ppr, positive predictivity; Spe, specificity; Acc, accuracy; F_1_, F_1_ score.

### 3.3 The WSL Setting

The WSL model is trained on the five coarsely-annotated datasets from the PhysioNet/CinC challenge. MITBIH-AR-DS1 is used as the validation set, and the other three finely-annotated datasets are used as the test sets. For ease of batch processing during the model training, all recordings in the training set are padded or truncated at the end to 20 s. The recordings in the validation set are also split into segments of 20 s.

**FIGURE 5:**
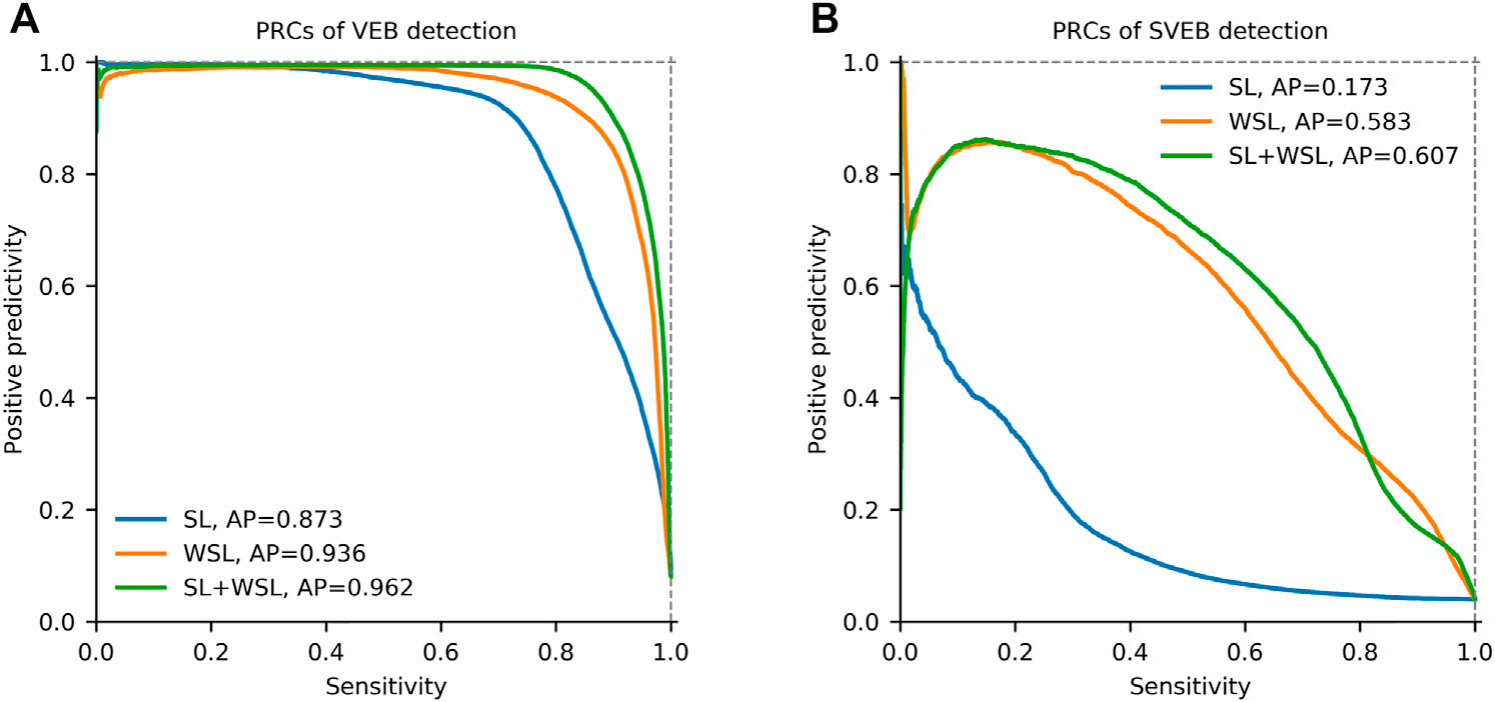
Precision-recall curves (PRCs) of the detection for ventricular ectopic beats (VEB) and supraventricular ectopic beats (SVEB) on the total dataset in different learning settings. **(A)** PRCs of VEB detection. **(B)** PRCs of SVEB detection. SL = supervised learning. WSL = weakly supervised learning. AP = average precision.

The evaluation results on the test sets are shown in Table 3. By comparing the scores with those in the SL setting, we find that the WSL model improves the scores for detecting SVEB, VEB and N on all of the test sets. Especially, the scores for SVEB detection are improved most significantly. For example, on the dataset of MITBIH-AR-DS2, the *Sen*, *Ppr*, and *F*_1_ scores for SVEB detection are improved from 0.066, 0.286, and 0.108 to 0.806, 0.799, and 0.803, respectively. The differences are shown visually by the PRCs in Figure 5. In the detection of both VEB (Figure 5A) and SVEB (Figure 5B), the curves for the WSL model (in orange) cover significantly larger areas than that for the SL model (in blue), especially in the detection of SVEB.

### 3.4 The SL + WSL Setting

In this two-stage training setting, the model is pre-trained in SL for 10 epochs on the segments from the first half of each recording in the MITBIH-AR-DS1. Then the model is trained in WSL on the same data in the WSL setting, with the last-half recordings in the MITBIH-AR-DS1 as the validation set. Finally, the model is tested on the three test sets, with the results shown in Table 3. These results are very close to that in the WSL setting, which is also shown in Figure 5. The WSL and SL + WSL models have superiority over each other in different datasets and metrics. For example, the WSL model outperforms the SL + WSL model in *Ppr* and *F*_1_ for detecting SVEB on the INCART, whereas the SL + WSL model outperforms the WSL model in *Sen* and *F*_1_ for detecting SVEB on the MITBIH-SUP. On the total test set, the overall performance (indicated by *F*_1_ and *AP*) of the SL + WSL model is superior to that of the WSL model in detecting both SVEB and VEB. Especially, in the detection of SVEB, the SL + WSL model achieves obvious better scores (*F*_1_ of 0.610, *AP* of 0.601) than the WSL model (*F*_1_ of 0.550, *AP* of 0.583) on the total test set.

### 3.5 Stability Assessment

**FIGURE 6:**
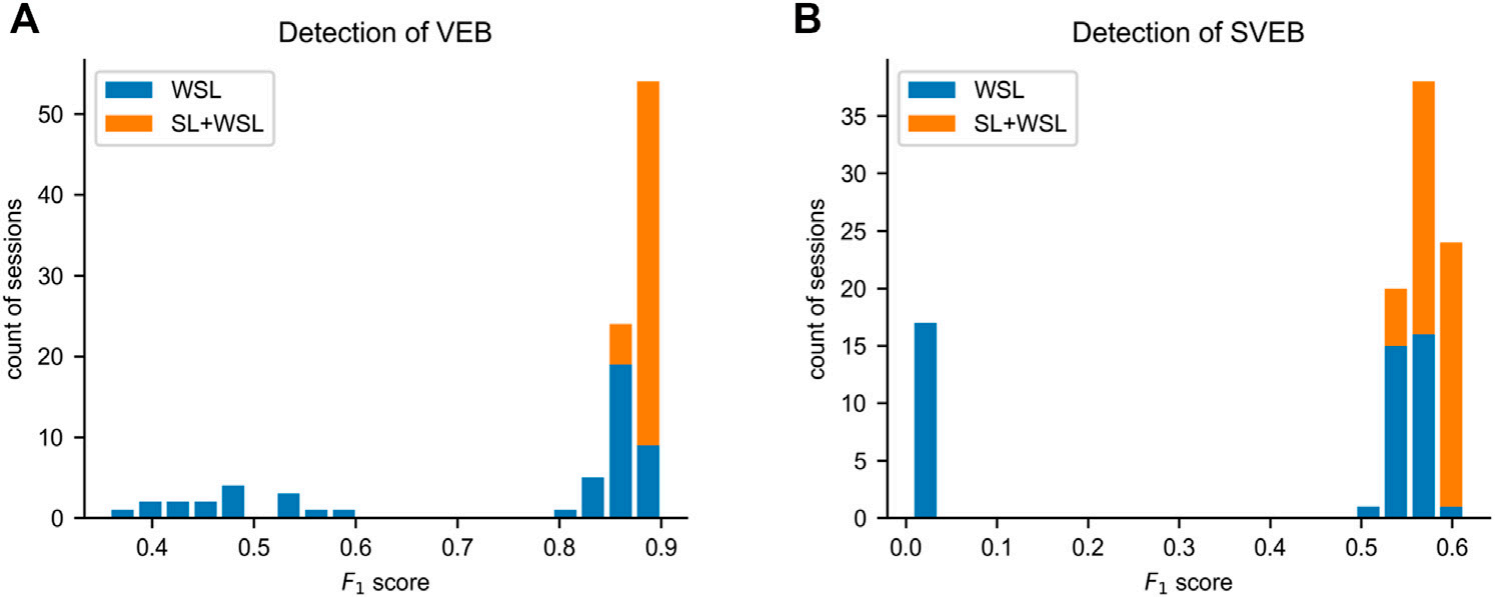
Distributions of the *F*_1_ scores on the total test data in multiple independent training sessions. **(A)** The histogram of the results for ventricular ectopic beat (VEB) detection. **(B)** The histogram of the results for supraventricular ectopic beat (SVEB) detection. SL = supervised learning. WSL = weakly supervised learning.

Due to the ill-posed problem of WSL, there is a chance that the rules learned by a WSL model deviate from the ground truth. To assess the stability of the model performance from training to training, we train the model independently 50 times in each of the WSL and SL + WSL settings. In the WSL setting, the *F*_1_ scores for SVEB detection and VEB detection are 0.370 ± 0.260 and 0.740 ± 0.187, respectively, whereas in the SL + WSL setting, the *F*_1_ scores for SVEB detection and VEB detection are 0.582 ± 0.019 and 0.886 ± 0.009, respectively. The distribution of the scores on the total test data are shown by the histograms in Figure 6. The results show that the performance of the WSL model in detecting both arrhythmias fluctuates wildly from training to training. The distribution of the scores for SVEB detection is polarized: some scores are clustered at the top pole, while other scores are located near the bottom pole. This suggests that different hypotheses of the detection model can satisfy the weak constraints of the recording labels and cause the instability of the model performance from training to training. By contrast, the performance of the SL + WSL model is very stable in multiple training sessions. The SL-based pre-training only initializes the model with a few training samples. It indicates that proper initialization can avoid the unstable performance of a WSL model.

### 3.6 Ablation Studies

Several ablation studies are conducted to evaluate the effects of the proposed knowledge-based features, DNN-based feature extraction and masked aggregation on the performance of the WSDL-AD framework. And all of the ablation studies are in the SL + WSL setting.

#### 3.6.1 Knowledge-Based Features

**FIGURE 7:**
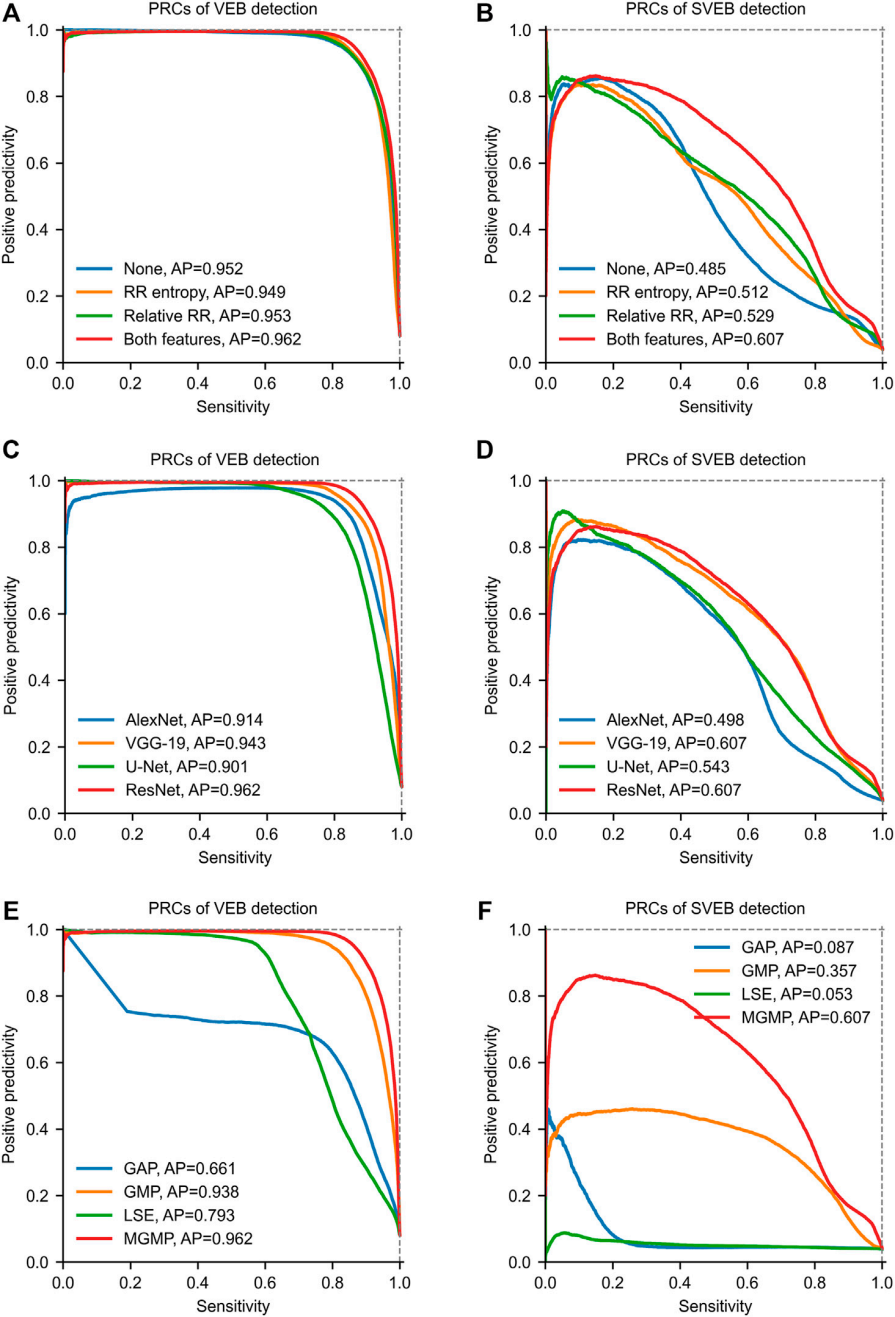
Precision-recall curves (PRCs) of the ablation studies on the total test data. **(A)** and **(B)** show PRCs of models with different knowledge-based features. **(C)** and **(D)** show PRCs of models with different feature extraction networks. **(E)** and **(F)** show PRCs of models with different aggregation mechanisms. GAP = global average pooling. GMP = global max pooling. LSE = Log-Sum-Exp. MGMP = masked global max pooling. AP = average precision. The parameter *b* of LSE is set to 5.

Ablation experiments are conducted to evaluate the contributions of knowledge-based features to the prediction. Four configurations are studied in the experiments, including *none* (using only DNN features), *RR entropy* (using DNN features and RR entropy), *relative RR interval* (using DNN features and relative RR interval), *both features* (using DNN features and both of the knowledge-based features). The test results on the total test data are shown by PRCs in Figures 7A,B. The PRCs of VEB detection are very similar among different configurations, so the knowledge-based features have little effect on the VEB detection. In contrast, there are significant differences among the PRCs for the SVEB detection. The model with no knowledge-based features has an AP score of 0.485, while applying the RR entropy and relative RR interval alone improves the score to 0.512 and 0.529 respectively. The joint application of both knowledge-based features further improves the score to 0.607. Therefore, the knowledge-based features have positive effects on the model’s performance in detecting SVEB.

#### 3.6.2 DNN-Based Feature Extraction Methods

In our framework, the DNN-based features are extracted using the ResNet. But our framework is also compatible with other kinds of networks for feature extraction. To evaluate the impacts of the network structure on the model performance, we conduct ablation experiments with different types of networks for feature extraction. Besides the ResNet, several well-known networks are tested for comparison, including AlexNet (Krizhevsky et al., 2012), VGG-19 (Simonyan and Zisserman, 2014) and U-Net (Ronneberger et al., 2015). Since these networks are all originally designed for processing 2D images, we replace the 2D convolutional and pooling layers with their 1D counterparts to adapt to the processing of 1D ECG signals. The recommended hyperparameters of these networks are adopted in our experiments. Some dimension-related hyperparameters (such as, convolutional kernel size and pool size) are converted to the 1D counterparts. For example, the convolutional kernel size (3 × 3) of the VGG-19 is converted to 3. The test results on the total test set are shown in Figures 7C,D. From the results, we can find that the performances of models with different network structures are obviously different. Among these networks, ResNet achieves the best performance in both SVEB (*AP* = 0.607) and VEB (*AP* = 0.962) detections on the total test data. The performance of VGG-19 is very close to that of ResNet, and the main difference lies in the detection of VEB (*AP* = 0.943). By contrast, the performances of models with AlexNet and U-Net are much lower than that of ResNet. These results indicate that the structure of the feature-extraction network has important impacts on the model performance. To obtain good performance, it is necessary to choose a proper network structure for feature extraction.

#### 3.6.3 Aggregation Mechanisms

Four aggregation mechanisms are compared in our ablation studies, including GAP, GMP, LSE (*b* = 5), and MGMP. The test results on the total test data are shown in Figures 7E,F. In detecting VEB, GAP achieves the poorest performance (AP = 0.661) among all of the tested mechanisms, which may be attributed to the fact that an arrhythmia epoch may only account for a small portion of a recording. The performance of LSE (AP = 0.793) is better than GAP since it makes a compromise between GAP and GMP. Much better performances are achieved by GMP (AP = 0.938) and MGMP (AP = 0.962). It may be because that these two mechanisms are consistent with the basic principle that an arrhythmia should be included in the global prediction as long as it exists somewhere in the recording. As for the detection of SVEB, the performances of GMP and MGMP are also significantly better than GAP and LSE. Especially, the MGMP, achieving an AP of 0.607, outperforms all other mechanisms by a large margin. This indicates that the masked mechanism is indeed helpful to improve the performance of WSL-based arrhythmia detection, especially for arrhythmias with subtle morphological changes, e.g., SVEB.

### 3.7 Analysis of Detection Examples

**FIGURE 8:**
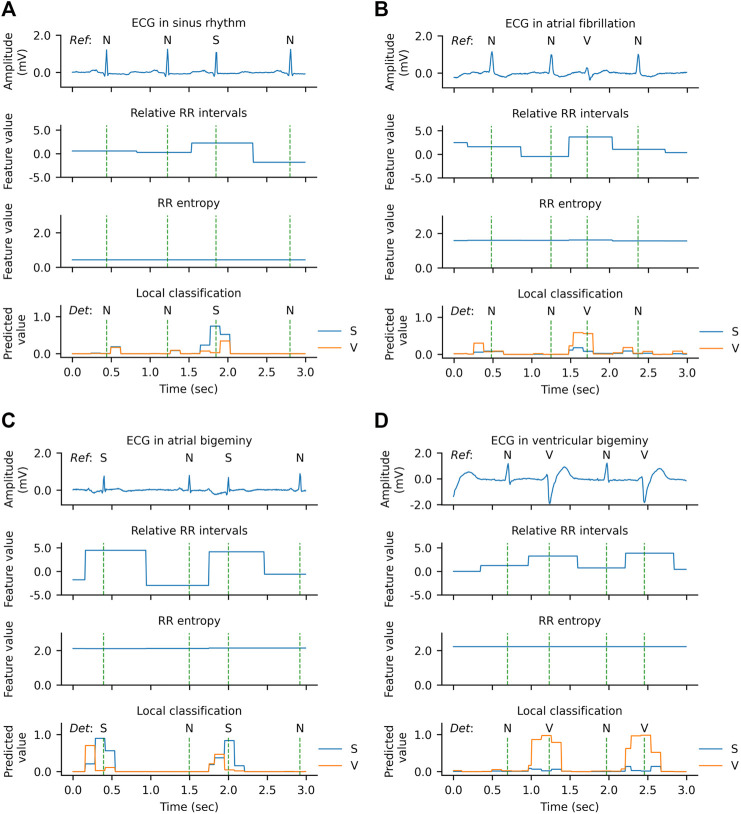
Qualitative results of arrhythmia detection on the MITBIH-AR-DS2 in the SL + WSL setting. **(A)** An example during normal rhythm. **(B)** An example during atrial fibrillation. **(C)** An example during atrial bigeminy. **(D)** An example during ventricular bigeminy. The vertical dashed lines indicate the positions of R peaks. In the ECG waveform charts, the reference category of each heartbeat is labeled above the ECG. In the local classification charts, the predictions for supraventricular ectopic beats (S) are drawn in blue, the predictions for ventricular ectopic beats (V) are drawn in orange, and the detected categories are labeled above the prediction lines. N denotes a normal or bundle branch block beat. Since the probabilities of N, S, and V add up to one at each sampling point, the predictions for N are not plotted in the figure for clarity.

The qualitative results of the WSDL-AD model in detecting SVEB and VEB are shown in Figure 8. In this figure, both the ECG signal and the knowledge-based feature maps (including relative RR interval and RR entropy) of each example are present. The local classifications contain the rhythm-wise probability distribution at each sampling point. The beat-by-beat detections are derived from the local predictions by selecting the predictions at the R peaks. The local classifications indicate that the WSL models have learned the ability to detect SVEBs and VEBs in various contextual rhythms, such as normal sinus rhythm, atrial fibrillation (AF), atrial bigeminy (AB), and ventricular bigeminy (VB). And, in most cases, the model has adequate confidence for the classification, where the predicted probability for some class is significantly higher than that for other classes. The value of the relative RR interval exhibits a positive correlation with the occurrence of SVEB and VEB, which is in line with our expectations and thus can serve as an effective indicator. However, a shortened RR interval, manifested by the high value of relative RR interval, doesn’t necessarily indicate an ectopic beat, because it may be caused by other arrhythmias, such as AF. The RR entropy, on the other hand, reflects the variation of RR intervals in the context: when the RR interval changes greatly (e.g., during AF, AB, or VB), the entropy value is at a high level, and vice versa (e.g., during sinus rhythm). Therefore, the joint application of relative RR interval and RR entropy can supply the information about the significance of a local RR-interval change, and help to increase the precision of the detection.

**FIGURE 9:**
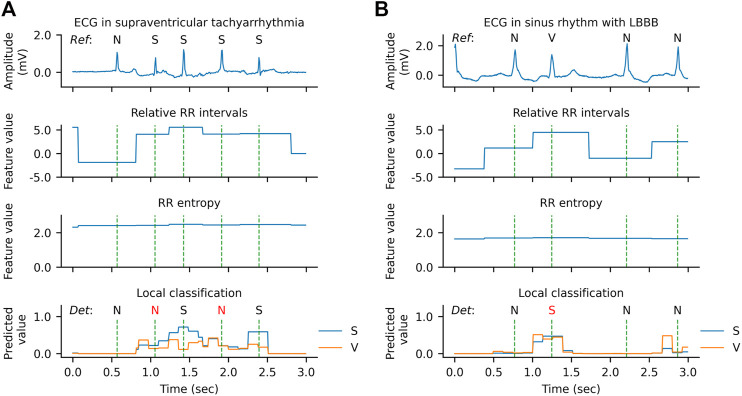
Qualitative results of error predictions. **(A)** An example of misclassifying supraventricular ectopic beats (S) as normal or bundle branch block beats (N) during supraventricular tachyarrhythmia. **(B)** An example of misclassifying a ventricular ectopic beat (V) as an S during left bundle branch block (LBBB).

However, there are also some cases where the model has low confidence in the classification or even makes errors, as shown in Figure 9. In the example of Figure 9A, two SVEBs during supraventricular tachyarrhythmia (SVTA) are misclassified as N. This can be attributed to the successive occurrence of SVEBs during SVTA, where the change of RR intervals from beat to beat is not significant, and especially most SVEBs are followed by noncompensatory pauses. Figure 9B presents an example that a VEB during the left bundle branch block (LBBB) is misclassified as SVEB. At the beat, the predicted probability for VEB is slightly less than that for SVEB, and both probabilities are significantly higher than that for N. It implies that the model has enough confidence to classify the beat as an ectopic beat, but has not enough confidence to distinguish whether it is an SVEB or VEB. One possible reason for this is that the QRS complex of the VEB is not significantly wider than that of the neighboring beats.

## 4 Discussion

In this study, we propose a WSL framework (WSDL-AD) for the beat-by-beat detection of arrhythmias, which requires only coarse-grained record-level annotations during the model training. The evaluation on independent datasets shows that a WSDL-AD model is able to learn the ability to detect VEB and SVEB from the coarsely annotated ECGs. In particular, our WSL model outperforms its SL counterpart by a large margin on multiple external test sets, which indicates that the WSL framework can facilitate the generalization ability of arrhythmia detection by exploring the large amount of coarsely-annotated ECG data. The biggest improvement is in the detection of SVEB, which is more difficult to detect because its waveform variation is usually very subtle. In this section, we will compare the results of our method with that of other state-of-the-art studies, and discuss the implications and limitations of this study.

### 4.1 Comparison With Other Studies

We compare our results with that of representative previous studies. The previous studies on the heartbeat classification can be categorized into two types: inductive learning (or induction), which learns general rules from labeled training samples and applies the rules to test samples; and transductive learning (or transduction), which learns the detection rules from both the labeled training samples and the unlabeled test samples, and applies the rules on the same test samples. This study and most previous studies are in inductive learning. Some studies that based on unsupervised domain adaptation are in transductive learning (Li et al., 2021; Wang et al., 2021). Although the models of transductive learning usually achieve better performance than inductive learning models, their requirement for the unlabeled target samples during the model training stage is hard to satisfy, since there are always new patients in routine clinical practice.

The MITBIH-AR-DS2 dataset is mostly used by previous studies for model evaluation, and the results of some representative studies are shown in Table 4. On the dataset, the test scores of our WSL models (in both WSL and SL + WSL settings) in detecting SVEB and VEB are significantly superior to that of the state-of-the-art methods of supervised inductive learning (Guo et al., 2019; Niu et al., 2020), and comparable to the state-of-the-art results of unsupervised transductive learning (Li et al., 2021; Wang et al., 2021). Compared with previous inductive models, the improvements of *F*_1_ scores are >8% (from 0.766 to 0.832) for SVEB and >4% (from 0.898 to 0.936) for VEB. The test results of previous studies on the MITBIH-SUP dataset and the INCART dataset are present in Tables 5,6 respectively. On the MITBIH-SUP dataset, our WSL models substantially outperform previous studies of induction (increasing the *F*_1_ scores by >290% for SVEB and >11% for VEB) and transduction methods (increasing the *F*_1_ scores by >66% for SVEB and >3% for VEB). Similar improvements are also observed for the INCART dataset. In particular, for the detection of SVEB, our WSL models have a big superiority over the transduction methods on these two datasets, although the transduction methods have optimized their models according to the test samples. These improvements indicate that the proposed WSL method can learn robust rules from a large amount of coarsely annotated data and has a better generalization ability.

**TABLE 4:** Comparison of our method with other studies on DS2 of the MITBIH-AR dataset.

Studies	Learning type	N			S			V		
		*Se*	*Ppr*	*F*_*1*_	*Se*	*Ppr*	*F*_*1*_	*Se*	*Ppr*	*F*_*1*_
De Chazal et al. (2004)	Induction	0.869	0.992	0.926	0.759	0.385	0.511	0.777	0.819	0.794
Llamedo and Martínez. (2010)	Induction	0.776	**0.995**	0.872	0.765	0.413	0.536	0.829	0.880	0.854
Mar et al. (2011)	Induction	0.896	0.991	0.841	0.832	0.335	0.478	0.868	0.759	0.809
Zhang et al. (2014)	Induction	0.889	0.990	0.937	0.791	0.360	0.495	0.855	0.928	0.890
Raj and Ray. (2018)	Induction	0.909	0.994	0.950	0.808	0.488	0.608	0.822	0.854	0.838
Garcia et al. (2017)	Induction	0.940	0.980	0.959	0.620	0.530	0.571	0.873	0.594	0.707
Guo et al. (2019)	Induction	—	—	—	0.627	0.612	0.619	0.913	0.883	0.898
Niu et al. (2020)	Induction	0.989	0.974	0.981	0.765	0.766	0.766	0.857	0.941	0.897
Wang et al. (2021)	Transduction	0.991	0.984	0.990	0.765	0.902	0.830	**0.940**	0.923	0.930
Li et al. (2021)	Transduction	**0.994**	0.983	0.989	0.772	**0.934**	**0.845**	0.906	0.944	0.924
This work (WSL setting)	Induction	0.990	0.987	0.988	0.806	0.799	0.803	0.902	0.950	0.925
This work (SL + WSL setting)	Induction	0.990	0.992	**0.991**	**0.886**	0.785	0.832	0.916	**0.956**	**0.936**

N, normal or bundle branch block beat; S, supraventricular ectopic beat; V, ventricular ectopic beat; Se, sensitivity; Ppr, positive predictivity; F_1_, F_1_ score.

The bold text indicates the maximum of each column.

**TABLE 5:** Comparison of our method with other studies on the MITBIH-SUP dataset.

Studies	Learning type	S			V		
		*Se*	*Ppr*	*F*_*1*_	*Se*	*Ppr*	*F*_*1*_
Al Rahhal et al. (2016)	Induction	0.088	0.143	0.109	0.652	0.093	0.163
Guo et al. (2019)	Induction	0.079	0.645	0.141	**0.868**	0.588	0.701
Wang et al. (2021)	Transduction	0.236	0.539	0.33	0.844	0.563	0.68
Li et al. (2021)	Transduction	0.238	0.472	0.316	0.785	0.724	0.753
This work (WSL setting)	Induction	0.325	**0.776**	0.458	0.803	**0.774**	**0.788**
This work (SL + WSL setting)	Induction	**0.452**	0.705	**0.551**	0.795	0.768	0.782

S, supraventricular ectopic beat; V, ventricular ectopic beat; Se, sensitivity; Ppr, positive predictivity; F_1_, F_1_ score.

The bold text indicates the maximum of each column.

**TABLE 6:** Comparison of our method with other studies on the INCART dataset.

Studies	Learning type	S			V		
		*Se*	*Ppr*	*F*_*1*_	*Se*	*Ppr*	*F*_*1*_
Llamedo and Martínez. (2010)	Induction	0.77	0.39	0.52	0.81	0.87	0.84
Al Rahhal et al. (2016)	Induction	0.156	0.025	0.04	0.751	0.376	0.501
Wang et al. (2021)	Transduction	0.711	0.435	0.54	**0.901**	0.903	0.90
This work (WSL setting)	Induction	0.852	**0.575**	**0.687**	0.886	0.950	0.917
This work (SL + WSL setting)	Induction	**0.927**	0.519	0.665	0.880	**0.976**	**0.926**

N, normal or bundle branch block beat; S, supraventricular ectopic beat; V, ventricular ectopic beat; Se, sensitivity; Ppr, positive predictivity; F_1_, F_1_ score.

The bold text indicates the maximum of each column.

### 4.2 Implications of the Proposed Method

This study shows that the WSL approach is effective to improve the generalization ability of beat-by-beat arrhythmia detectors by leveraging the large amounts of coarsely-annotated ECG data. An arrhythmia detector with both fine detection granularity and good generalization ability has important implications for clinical practice. The fine granularity is necessary for measuring the burden and pattern (e.g., bigeminy and trigeminy) of arrhythmias, which can be used to assess critical risks (e.g., stroke and heart failure) in clinic (Boriani et al., 2014; Marcus, 2020). On the other hand, the generalization ability is a necessary prerequisite for an algorithm to be trusted for clinical use, because ECG is susceptible to environmental and individual differences. The diagnoses made by currently used algorithms still need to be reviewed by doctors. However, with the explosion of ECG data from mobile devices, it is not practical to rely on doctors to review every record. With the improvements in generalization ability, our WSL method can deal with more situations and make more reliable detections independently, which are of significance to prompt the revolution of automatic ECG diagnosis.

This superiority of the WSL models may source from the fact that their training data are from a much larger patient group than that of the SL models. The coarse-grained annotations usually take much less time and effort than fine-grained annotations. And to ensure the correctness of the annotations, fine annotation needs multiple experts to reach a consensus on the labels of each beat, while coarse annotation only needs a consensus on the global labels. In addition, in many medical institutions, the electronic medical records, containing both physiological signals and diagnostic reports, are inherent coarsely annotated data and could be used to train the WSL models. Therefore, the amount of coarsely annotated data could be accumulated rapidly in the future, which provides essential substrates for continually improving the reliability of automatic arrhythmia detectors.

As demonstrated by the ablation experiments, the proposed knowledge-based features and masked aggregation mechanism also play an important role in improving the performance of the WSL models. One of the advantages of the knowledge-based features is that they integrate the information in a very wide context, which is usually difficult to learn by the model self, especially under weak supervision. By fusing the knowledge-based features and the DNN-extracted features, the representative ability of the feature vector might be enhanced, thus causing the detection performance to be improved. On the other hand, the masked aggregation mechanism selects the representative prediction for each heartbeat at the specified reference point, which greatly reduces the space of possible local predictions. Besides, because the reference points belong to the same type of ECG subwave, e.g., the R peak, the features aligned to the reference points are also semantic comparable with each other. Consequently, the masked aggregation mechanism helps to improve the performance of ectopic beat detection. Furthermore, the results of multiple independent training sessions reveal the instability of the training process of WSDL-AD. We also demonstrate that SL-based pre-training on a few finely-annotated samples can effectively improve the stability of the training process. It implies that the initialization has a critical effect on the training process of the WSL model. These methods proposed in this study may also be enlightening to further studies in arrhythmia detection and even other fields.

The computational complexity of our framework can be divided into several parts, which are corresponding to preprocessing, QRS complexes detection, knowledge-based features extraction, DNN-based features extraction, local prediction, and aggregation, respectively. Among these parts, the part of DNN-based features extraction dominates the computational complexity of the framework. The network for feature extraction is a 1D ResNet whose computational complexity mainly comes from the convolutional layers in it. The computational complexity of each convolutional layer is O(*K*×*M*×*N*), where *K* is the kernel number, *M* is the kernel size (kernel length × channels), and *N* is the signal length. By putting the hyperparameters in the formula, we get O(*K*×*M*×*N*) = O (32 × 16×32×*N*) = O(*N*). There are only nine convolutional layers in our 1D ResNet, and the feature maps are gradually down-sampled. Taken together, the computational complexity of the 1D ResNet is O (9 × 32×16 × 32×*N*) = O(*N*). Thus, the computational complexity of the 1D ResNet is linear to the signal length. Besides, the number of layers and the number of convolutional kernels in our network are much smaller than that of other well-known networks, such as VGG-19 (Krittanawong et al., 2019) and U-Net (Ronneberger et al., 2015). Therefore, the computational complexity of our framework is moderate, which is critical for scenarios where computing resources are scarce, such as mobile ECG monitoring.

### 4.3 Limitations

This work also has some limitations. First, the WSL models have a high error rate for SVEB detection. The waveform patterns of SVEB are usually subtle and therefore difficult to be recognized by the model. To address this problem, collecting more training data or improving the design of the WSL framework (e.g., extracting features of the P wave) would be helpful. Second, the WSDL-AD framework is not evaluated for detecting other kinds of ECG abnormalities, such as branch bundle blocks and ST segment changes. Future work is required to assess the effectiveness of WSL in detecting more diverse ECG abnormalities.

## 5 Conclusion

In conclusion, this study develops and evaluates a WSDL framework for beat-by-beat arrhythmia detection, by which we demonstrate the feasibility of training a fine-grained arrhythmia detector on only coarsely-labeled ECG data. The evaluations on multiple external datasets show that the proposed framework has a significant superiority in generalization ability over previous SL-based methods. The knowledge-based features and masked aggregation mechanism also have important contributions to the performance of the model, while the SL-based pre-training helps to improve the stability of the training process. Furthermore, the computational complexity of our framework is moderate, which permits the models to be deployed on hardware with limited computing resources. Our approach would substantially reduce the burden of data annotation and enhance the reliability of beat-by-beat arrhythmia detection, and therefore has a great potential to promote the application of automatic cardiac monitoring both in and out of hospitals.

## Data Availability

The original contributions presented in the study are included in the article/supplementary material, further inquiries can be directed to the corresponding authors.

## Footnotes

1 MITBIH-AR-DS1 comprises recordings 101, 106, 108, 109, 112, 114, 115, 116, 118, 119, 122, 124, 201, 203, 205, 207, 208, 209, 215, 220, 223, and 230.

2 MITBIH-AR-DS2 comprises recordings 100, 103, 105, 111, 113, 117, 121, 123, 200, 202, 210, 212, 213, 214, 219, 221, 222, 228, 231, 232, 233, and 234.

## References

[B1] AAMI (2012). “Testing and Reporting Performance Results of Cardiac Rhythm and ST Segment Measurement Algorithms,” in *ANSI/AAMI EC57.* Association for the Advancement of Medical Instrumentation.

[B2] Baman T. S. Lange D. C. Ilg K. J. Gupta S. K. Liu T.-Y. Alguire C. (2010). Relationship between burden of Premature Ventricular Complexes and Left Ventricular Function. Heart Rhythm 7 (7), 865–869. 10.1016/j.hrthm.2010.03.036 20348027

[B3] Binici Z. Intzilakis T. Nielsen O. W. Køber L. Sajadieh A. (2010). Excessive Supraventricular Ectopic Activity and Increased Risk of Atrial Fibrillation and Stroke. Circulation 121 (13), 1904–1911. 10.1161/CIR.0b013e3181f3321810.1161/CIRCULATIONAHA.109.874982 20404258

[B4] Boriani G. Glotzer T. V. Santini M. West T. M. De Melis M. Sepsi M. (2014). Device-detected Atrial Fibrillation and Risk for Stroke: an Analysis of >10 000 Patients from the SOS AF Project (Stroke preventiOn Strategies Based on Atrial Fibrillation Information from Implanted Devices). Eur. Heart J. 35 (8), 508–516. 10.1093/eurheartj/eht491 24334432 PMC3930873

[B5] Chen Z. (2003). Assessing Sequence Comparison Methods with the Average Precision Criterion. Bioinformatics 19 (18), 2456–2460. 10.1093/bioinformatics/btg349 14668231

[B6] Choe J. Oh S. J. Lee S. Chun S. Akata Z. Shim H. (2020). “Evaluating Weakly Supervised Object Localization Methods Right,” in Proceedings of the IEEE/CVF Conference on Computer Vision and Pattern Recognition, 3133–3142.

[B7] deChazal P. O'Dwyer M. Reilly R. B. (2004). Automatic Classification of Heartbeats Using ECG Morphology and Heartbeat Interval Features. IEEE Trans. Biomed. Eng. 51 (7), 1196–1206. 10.1109/tbme.2004.827359 15248536

[B8] Degirmenci M. Ozdemir M. A. Izci E. Akan A. (2021). Arrhythmic Heartbeat Classification Using 2D Convolutional Neural Networks. Irbm. 10.1016/j.irbm.2021.04.002

[B9] Deyell M. W. Park K.-M. Han Y. Frankel D. S. Dixit S. Cooper J. M. (2012). Predictors of Recovery of Left Ventricular Dysfunction after Ablation of Frequent Ventricular Premature Depolarizations. Heart Rhythm 9 (9), 1465–1472. 10.1016/j.hrthm.2012.05.019 22640894

[B10] Donnelly K. (2006). SNOMED-CT: The Advanced Terminology and Coding System for eHealth. Stud. Health Technol. Inform. 121, 279–290. 17095826

[B11] Garcia G. Moreira G. Menotti D. Luz E. (2017). Inter-patient ECG Heartbeat Classification with Temporal VCG Optimized by PSO. Sci. Rep. 7 (1), 10543–10611. 10.1038/s41598-017-09837-3 28874683 PMC5585360

[B12] Golany T. Radinsky K. (2019). “Pgans: Personalized Generative Adversarial Networks for Ecg Synthesis to Improve Patient-specific Deep Ecg Classification,” in Proceedings of the AAAI Conference on Artificial Intelligence, 557–564.

[B13] Goldberger A. L. Amaral L. A. Glass L. Hausdorff J. M. Ivanov P. C. Mark R. G. (2000). PhysioBank, PhysioToolkit, and PhysioNet: Components of a New Research Resource for Complex Physiologic Signals. Circulation 101 (23), e215–20. 10.1161/01.cir.101.23.e215 10851218

[B14] Greenwald S. D. Patil R. S. Mark R. G. (1990). “Improved Detection and Classification of Arrhythmias in Noise-Corrupted Electrocardiograms Using Contextual Information,” in Computers in Cardiology (Chicago, IL. 1562826

[B15] Guo L. Sim G. Matuszewski B. (2019). Inter-patient ECG Classification with Convolutional and Recurrent Neural Networks. Biocybernetics Biomed. Eng. 39 (3), 868–879. 10.1016/j.bbe.2019.06.001

[B16] He K. Zhang X. Ren S. Sun J. (2016). “Deep Residual Learning for Image Recognition,” in IEEE/CVF Conference on Computer Vision and Pattern Recognition, 770–778.

[B17] He K. Zhang X. Ren S. Sun J. (2015). “Delving Deep into Rectifiers: Surpassing Human-Level Performance on Imagenet Classification,” in International Conference on Computer Vision, 1026–1034.

[B18] He R. Liu Y. Wang K. Zhao N. Yuan Y. Li Q. (2020). Automatic Detection of QRS Complexes Using Dual Channels Based on U-Net and Bidirectional Long Short-Term Memory. IEEE J. Biomed. Health Inform. PP (4), 1052–1061. 10.1109/JBHI.2020.3018563 32822314

[B19] Ioffe S. Szegedy C. (2015). “Batch Normalization: Accelerating Deep Network Training by Reducing Internal Covariate Shift,” in International conference on machine learning, 448–456.

[B20] Kingma D. P. Ba J. (2014). Adam: A Method for Stochastic Optimization. *arXiv preprint* .

[B21] Kiranyaz S. Ince T. Gabbouj M. (2016). Real-time Patient-specific ECG Classification by 1-D Convolutional Neural Networks. IEEE Trans. Biomed. Eng. 63 (3), 664–675. 10.1109/tbme.2015.2468589 26285054

[B22] Kolesnikov A. Lampert C. H. (2016). “Seed, Expand and Constrain: Three Principles for Weakly-Supervised Image Segmentation,” in European Conference on Computer Vision, 695–711.

[B23] Krittanawong C. Johnson K. W. Rosenson R. S. Wang Z. Aydar M. Baber U. (2019). Deep Learning for Cardiovascular Medicine: a Practical Primer. Eur. Heart J. 40 (25), 2058–2073. 10.1093/eurheartj/ehz056 30815669 PMC6600129

[B24] Krizhevsky A. Sutskever I. Hinton G. E. (2012). “Imagenet Classification with Deep Convolutional Neural Networks,” in Adv. Neur. Inf. Process. Syst., 1097–1105.

[B25] Li J. Wang G. Chen M. Ding Z. Yang H. (2021). Mixup Asymmetric Tri-training for Heartbeat Classification under Domain Shift. IEEE Signal. Process. Lett. 28, 718–722. 10.1109/lsp.2021.3066068

[B26] Li Y. Pang Y. Wang J. Li X. (2018). Patient-specific ECG Classification by Deeper CNN from Generic to Dedicated. Neurocomputing 314, 336–346. 10.1016/j.neucom.2018.06.068

[B27] Liu F. Liu C. Zhao L. Zhang X. Wu X. Xu X. (2018). An Open Access Database for Evaluating the Algorithms of Electrocardiogram Rhythm and Morphology Abnormality Detection. J Med. Imaging Hlth Inform. 8 (7), 1368–1373. 10.1166/jmihi.2018.2442

[B28] Llamedo M. Martinez J. P. (2010). Heartbeat Classification Using Feature Selection Driven by Database Generalization Criteria. IEEE Trans. Biomed. Eng. 58 (3), 616–625. 10.1109/TBME.2010.2068048 20729162

[B29] Lu Y. Jiang M. Wei L. Zhang J. Wang Z. Wei B. (2021). Automated Arrhythmia Classification Using Depthwise Separable Convolutional Neural Network with Focal Loss. Biomed. Signal Process. Control. 69, 102843. 10.1016/j.bspc.2021.102843

[B30] Mar T. Zaunseder S. Martínez J. P. Llamedo M. Poll R. (2011). Optimization of ECG Classification by Means of Feature Selection. IEEE Trans. Biomed. Eng. 58 (8), 2168–2177. 10.1109/tbme.2011.2113395 21317067

[B31] Marcus G. M. (2020). Evaluation and Management of Premature Ventricular Complexes. Circulation 141 (17), 1404–1418. 10.1161/circulationaha.119.042434 32339046

[B32] Moody G. B. Mark R. G. (2001). The Impact of the MIT-BIH Arrhythmia Database. IEEE Eng. Med. Biol. Mag. 20 (3), 45–50. 10.1109/51.932724 11446209

[B33] Nair V. Hinton G. E. (2010). “Rectified Linear Units Improve Restricted Boltzmann Machines,” in International Conference on Machine Learning, 807–814.

[B34] Niu J. Tang Y. Sun Z. Zhang W. (2020). Inter-patient ECG Classification with Symbolic Representations and Multi-Perspective Convolutional Neural Networks. IEEE J. Biomed. Health Inform. 24 (5), 1321–1332. 10.1109/jbhi.2019.2942938 31545750

[B35] Ozdemir M. A. Ozdemir G. D. Guren O. (2021). Classification of COVID-19 Electrocardiograms by Using Hexaxial Feature Mapping and Deep Learning. BMC Med. Inform. Decis. Mak 21 (1), 170. 10.1186/s12911-021-01521-x 34034715 PMC8146190

[B36] Pathak D. Shelhamer E. Long J. Darrell T. (2014). “Fully Convolutional Multi-Class Multiple Instance Learning,” in International Conference on Learning Representations.

[B37] Perez Alday E. A. Gu A. Shah A. J. Robichaux C. Wong A. I. Liu C. (2020). Classification of 12-lead ECGs: the Physionet/computing in Cardiology challenge 2020. Physiol. Meas. 41 (12). Article 124003. 10.1088/1361-6579/abc960 PMC8015789 33176294

[B38] Pinheiro P. O. Collobert R. (2015). “From Image-Level to Pixel-Level Labeling with Convolutional Networks,” in IEEE/CVF Conference on Computer Vision and Pattern Recognition, 1713–1721.

[B39] Rahhal M. M. A. Bazi Y. AlHichri H. Alajlan N. Melgani F. Yager R. R. (2016). Deep Learning Approach for Active Classification of Electrocardiogram Signals. Inf. Sci. 345, 340–354. 10.1016/j.ins.2016.01.082

[B40] Raj S. Ray K. C. (2018). Sparse Representation of ECG Signals for Automated Recognition of Cardiac Arrhythmias. Expert Syst. Appl. 105, 49–64. 10.1016/j.eswa.2018.03.038

[B41] Rajan D. Thiagarajan J. J. (2018). “A Generative Modeling Approach to Limited Channel ECG Classification,” in Annual International Conference of the IEEE Engineering in Medicine and Biology Society, 2571–2574. 10.1109/EMBC.2018.8512757 30440933

[B42] Reyna M. S. NadiGu A. Alday P. Andres E. Liu C. Seyedi S. (2021). Will Two Do? Varying Dimensions in Electrocardiography: The PhysioNet - Computing in Cardiology Challenge 2021. Online. PhysioNet: PhysioNet. AvailableAccessed Feb. 25 2021]. 10.13026/jz9p-0m02

[B43] Richman J. S. Moorman J. R. (2000). Physiological Time-Series Analysis Using Approximate Entropy and Sample Entropy. Am. J. Physiology-Heart Circulatory Physiol. 278 (6), H2039–H2049. 10.1152/ajpheart.2000.278.6.h2039 10843903

[B44] Roger V. L. Go A. S. Lloyd-Jones D. M. Benjamin E. J. Berry J. D. Borden W. B. (2012). Executive Summary: Heart Disease and Stroke Statistics--2012 Update: a Report from the American Heart Association. Circulation 125 (22), 188–197. 10.1161/CIR.0b013e3182456d46 22215894

[B45] Ronneberger O. Fischer P. Brox T. (2015). “U-net: Convolutional Networks for Biomedical Image Segmentation,” in 18th International Conference on Medical Image Computing and Computer-Assisted Intervention (MICCAI), 234–241.

[B46] Sana F. Isselbacher E. M. Singh J. P. Heist E. K. Pathik B. Armoundas A. A. (2020). Wearable Devices for Ambulatory Cardiac Monitoring. J. Am. Coll. Cardiol. 75 (13), 1582–1592. 10.1016/j.jacc.2020.01.046 32241375 PMC7316129

[B47] Simonyan K. Zisserman A. (2014). Very Deep Convolutional Networks for Large-Scale Image Recognition. *arXiv preprint*.

[B48] Siontis K. C. Noseworthy P. A. Attia Z. I. Friedman P. A. (2021). Artificial Intelligence-Enhanced Electrocardiography in Cardiovascular Disease Management. Nat. Rev. Cardiol. 18 (7), 465–478. 10.1038/s41569-020-00503-2 33526938 PMC7848866

[B49] Srivastava N. Hinton G. Krizhevsky A. Sutskever I. Salakhutdinov R. (2014). Dropout: a Simple Way to Prevent Neural Networks from Overfitting. J. Mach. Learn. Res. 15 (1), 1929–1958.

[B50] Wagner P. Strodthoff N. Bousseljot R. D. Kreiseler D. Lunze F. I. Samek W. (2020). PTB-XL, a Large Publicly Available Electrocardiography Dataset. Sci. Data 7 (1), 154–215. 10.1038/s41597-020-0495-6 32451379 PMC7248071

[B51] Wang G. Chen M. Ding Z. Li J. Yang H. Zhang P. (2021). Inter-patient ECG Arrhythmia Heartbeat Classification Based on Unsupervised Domain Adaptation. Neurocomputing 454, 339–349. 10.1016/j.neucom.2021.04.104

[B52] Wulan N. Wang W. Sun P. Wang K. Xia Y. Zhang H. (2020). Generating Electrocardiogram Signals by Deep Learning. Neurocomputing 404, 122–136. 10.1016/j.neucom.2020.04.076

[B53] Zhai X. Zhou Z. Tin C. (2020). Semi-supervised Learning for ECG Classification without Patient-specific Labeled Data. Expert Syst. Appl. 158, 113411. 10.1016/j.eswa.2020.113411

[B54] Zhang Z. Dong J. Luo X. Choi K.-S. Wu X. (2014). Heartbeat Classification Using Disease-specific Feature Selection. Comput. Biol. Med. 46, 79–89. 10.1016/j.compbiomed.2013.11.019 24529208

[B55] Zheng J. Zhang J. Danioko S. Yao H. Guo H. Rakovski C. (2020). A 12-lead Electrocardiogram Database for Arrhythmia Research Covering More Than 10,000 Patients. Sci. Data 7 (1), 48–8. 10.1038/s41597-020-0386-x 32051412 PMC7016169

[B56] Zhou B. Khosla A. Lapedriza A. Oliva A. Torralba A. (2016). “Learning Deep Features for Discriminative Localization,” in IEEE/CVF Conference on Computer Vision and Pattern Recognition, 2921–2929.

[B57] Zhou Z.-H. (2018). A Brief Introduction to Weakly Supervised Learning. Natl. Sci. Rev. 5 (1), 44–53. 10.1093/nsr/nwx106

